# Overweight worsens the metabolic presentation of type 1 diabetes mellitus in children

**DOI:** 10.3389/fendo.2025.1740046

**Published:** 2026-01-09

**Authors:** Eszter Muzslay, Vivien Herczeg, Rozália Ildikó Pusztai, Vilmos Zoltán Forstreuter, Dorina Soós, Johanna Takács, Péter Tóth-Heyn, Andrea Luczay

**Affiliations:** 1 Pediatric Center, Hungarian Academy of Sciences – Magyar Tudományos Akadémia (MTA) Center of Excellence, Semmelweis University, Budapest, Hungary; 2 Károly Rácz Conservative Medicine Division, Doctoral School, Semmelweis University, Budapest, Hungary; 3 Faculty of Medicine, Semmelweis University, Budapest, Hungary; 4 Department of Social Sciences, Faculty of Health Sciences, Semmelweis University, Budapest, Hungary

**Keywords:** obesity, overweight, pediatric diabetology, pediatric endocrinology, type 1 diabetes mellitus

## Abstract

**Introduction:**

The prevalence of both obesity and type 1 diabetes mellitus (T1DM) has increased globally over the last decades. Overweight and obesity affect individuals with T1DM and influence not only the autoimmune pathogenesis but also the long-term complications of T1DM. This study aimed to investigate the effect of excess body weight on the clinical presentation of T1DM.

**Methods:**

We conducted a retrospective, single-center cohort study at the largest pediatric endocrinology center in Hungary. Data were collected from children diagnosed with T1DM between 2014 and 2023. A total of 994 patients’ presentation parameters and anthropometric data were analyzed. Based on BMI Z-scores, patients were categorized into three groups (normal-weight, overweight, and obese). Metabolic parameters at diagnosis were compared between groups.

**Results:**

The combined ten-year prevalence of overweight and obesity was found to be 15.9%. Significant between-group differences were observed in pH (p=0.005), bicarbonate (HCO_3_^-^; p=0.018), partial carbon-dioxide (pCO_2_) pressure (p=0.018), and C-peptide levels (p< 0.001). Lower pH, pCO_2_ and HCO_3_^-^ levels were found among those who were overweight, and higher C-peptide levels in children with obesity. Diabetic ketoacidosis (DKA) and severe DKA were seen at a significantly higher rate among children with overweight and obesity (p=0.013; p< 0.001).

**Discussion:**

Children with overweight presented with more severe metabolic derangement at T1DM onset. Obese and overweight children have a higher risk of having DKA at presentation despite elevated C-peptide levels, which suggest a greater residual β-cell function. These findings support the hypothesis that other factors, such as chronic inflammation, may contribute to T1DM manifestation. Our findings highlight the impact of overweight and obesity on the clinical presentation of T1DM. In case of weight loss in obese and overweight children, more attention should be paid to the classical symptoms of T1DM.

## Introduction

An increase in the incidence and prevalence of type 1 diabetes mellitus (T1DM) in children was observed in the last decades (1–3). The incidence is increasing by an average of 3-4% per year, a rate that cannot be explained by genetic causes (4). Therefore, the underlying causes are considered to be environmental factors. The obesogenic environment and the presence of overweight and obesity might add to the increasing prevalence of T1DM (5–10).

Overweight and obesity present an international problem in childhood with a continuously increasing prevalence. Several definitions are used to describe overweight and obesity worldwide. The World Health Organization (WHO) determines the presence of overweight and obesity based on the BMI Z-score. Overweight is defined as a BMI Z-score greater than one, while a score above two indicates obesity (11).

Individuals with T1DM are not exempt from the obesity pandemic. According to the SWEET (Better control in Pediatric and Adolescent diabeteS: Working to crEate CEnTers of Reference) Registry, 31.8% of children with T1DM are affected with overweight or obesity (WHO’s definition was used) (12). The presence of overweight in T1DM increases cardiometabolic risk and worsens the chronic complications of T1DM. In addition, insulin resistance caused by obesity leads to higher insulin requirements and complicates achieving optimal glycemic control and weight management in T1DM (5).

Previous studies reported the effect of overweight on pathogenesis and long-term complications of T1DM, but not the possible acute complications seen at presentation of T1DM. Our study aimed to better understand the impact of overweight and obesity on the presentation of T1DM. Therefore, in our study we aimed to assess the metabolic parameters at the onset of T1DM in children based on their body mass index (BMI) status, and to compare these parameters between normal-weight, overweight, and obese children with T1DM.

## Materials and methods

### Study setting and data collection

Our single-center retrospective cohort study was performed at the Bókay Unit of the Pediatric Center, Semmelweis University, Budapest, Hungary. In Hungary, our center is the largest in pediatric T1DM care, with almost 25% of Hungarian pediatric T1DM patients. Data were collected from all children with diabetes mellitus (DM) who received insulin treatment and had at least one visit between 1st of January 2014, and 31st of December 2023, at the inpatient and/or outpatient Endocrinology and Diabetes Unit of the Pediatric Center, Semmelweis University. Clinical data and laboratory results were obtained from the university’s e-MedSolution software. All children with a form of diabetes other than T1DM and those who did not have anthropometric measurements at the first ambulatory control were excluded from our study.

### Recorded parameters and methodology

Patients’ sex and the following parameters at manifestation of T1DM were collected: age, pH, bicarbonate (HCO_3_^-^), partial pressure of carbon dioxide (pCO_2_), blood glucose level (BG), hemoglobin A_1c_ (HbA_1c_), and C-peptide. Anthropometric data (weight, height) were recorded at T1DM diagnosis and the first ambulatory visit (three months after T1DM diagnosis). Standardized SI units were used for all recorded parameters. The presence of diabetic ketoacidosis (DKA) and its severity were classified according to the latest International Society for Pediatric and Adolescent Diabetes (ISPAD) guidelines and can be seen in **Table 1** (13).

**Table 1:** ISPAD criteria to classify the severity of DKA at the manifestation of T1DM (13).

No DKA	7.3 ≤ pH	or	18 ≤ HCO_3_^-^ (mmol/l)
Mild DKA	7.2 ≤ pH < 7.3	or	10 ≤ HCO_3_^-^ (mmol/l) < 18
Moderate DKA	7.1 ≤ pH < 7.2	or	5 ≤ HCO_3_^-^ (mmol/l) < 10
Severe DKA	pH < 7.1	or	HCO_3_^-^ (mmol/l) < 5

### Anthropometric data

From patients’ weight (kg) and height (m), their body mass index (BMI) and BMI Z-scores, modified by age and sex, were also calculated based on the Hungarian population standards (14, 15). At T1DM manifestation, the assumed dehydration due to the nature of DKA affected weight and, therefore, BMI status. Three months after the diagnosis of T1DM, a steady-state condition is presumed regarding anthropometric parameters, and the initial dehydration is resolved. BMI Z-scores at the diagnosis of T1DM were compared with those obtained three months later. In the presence of a statistically significant difference between the two time points, the three-month measurements were used for subsequent analyses. Thereafter we defined groups based on the Z-score in two ways. First, we formed the groups of ‘normal’ (1≥BMI Z-score) and ‘children with overweight’ (1<BMI Z-score). After that, children with overweight were further divided into ‘overweight’ (1<BMI Z-score ≤ 2) and ‘obese’ (2<BMI Z-score) categories based on the WHO’s definitions as shown in **Table 2** (11). Underweight children were combined with their normal-weight peers.

**Table 2:** Classification of children based on WHO standards using BMI Z-scores (11).

BMI status:	Normal*	Overweight	Obese
BMI criteria	Z-score ≤ 1	1 < Z-score ≤ 2	2 < Z-score

*In our study, children with low BMI Z-scores were merged with children with normal Z-scores.

### Statistical analysis

A professional biostatistician carried out the statistical analysis. Regarding the number of outliers, non-parametric statistical tests were used. To compare BMI Z-scores at T1DM diagnosis and three months later, a Wilcoxon-signed-rank test was used. Comparisons were done between two subgroups (“normal” vs “children with overweight”) and also between three subgroups (“normal” vs “overweight” vs “obese”). The Mann-Whitney U test was used for comparisons of two subgroups with r effect size measurement, and the Kruskal-Wallis H test was used to compare three subgroups with Bonferroni correction for all pairwise comparisons and calculation of the epsilon-squared (ϵ^2^) effect size measurement. To compare proportions, the Pearson’s chi-square test of independence with compare column proportions (adjust p-values Bonferroni method) was used. The effects of age and sex were also examined during groups comparisons using stratification/layer method. For this, age groups were formed based on the international SWEET database (0-5.99; 6-11.99; 12-17.99 years) (16). The level of significance was set at 0.05. All statistical analysis were performed by using IBM SPSS 30.0.0.0 version. Visualization was created using IBM SPSS 30.0.0.0 and Microsoft Office PowerPoint.

## Results

### Demographics

During the studied period, 1693 patients with DM appeared at our center. 699 children were excluded because of missing anthropometric data (633 children) or diabetes forms other than T1DM (66 children). Overall, 994 patients’ records were analyzed (548 boys, 55.1%); detailed data are provided in **Table 3**.

**Table 3:** Characteristics of children included in our study.

Number of patients		994
Mean ± SD age at T1DM manifestation (years)		8.57 ± 4.26
Age min-max at T1DM manifestation (years)		0.5 – 18.33
Female sex, n (%)		446 (44.9)
pH at T1DM manifestation, mean ± SD		7.29 ± 0.15
HCO_3_^-^ at T1DM manifestation (mmol/l), mean ± SD		17.35 ± 7.57
DKA status at T1DM manifestation	No DKA, n (%)	550 (56.5)
	Mild DKA, n (%)	175 (18.0)
	Moderate DKA, n (%)	123 (12.6)
	Severe DKA, n (%)	126 (12.9)
pCO_2_ at T1DM manifestation (mmHg), mean ± SD		29.96 ± 9.8
BG at T1DM manifestation (mmol/l), mean ± SD		24.87 ± 9.86
HbA_1c_ at T1DM manifestation (%), mean ± SD		11.59 ± 2.48
C-peptide at T1DM manifestation (ng/ml), mean ± SD		0.6 ± 0.56

The ten-year prevalence of obesity was found to be 5.9%, the prevalence of overweight was 10.0%, whereas overweight and obesity together occurred in 15.9% of children with T1DM over the study period.

### Subgroup analysis

Patient subgroups were formed based on the three-month BMI Z-score data. BMI Z-scores at T1DM diagnosis were significantly lower than at three months (Z= -21.622; p<0.001, r=0.02). As stated in the Methods, lower Z-scores may be a result of initial dehydration and cannot be used for further analysis. Baseline characteristics of each subgroup are shown in **Tables 4** and **5**.

**Table 4:** Characteristics of the two subgroups regarding the parameters recorded at the presentation of T1DM.

Recorded parameter	Normal (n=776)	Children with overweight (n=146)	p	effect size (r)
Age (years); Med [IR]	8.71 [4.83;11.98]	9.71 [6.23; 12.5]	**0.041**	0.067
pH; Med [IR]	7.35 [7.23; 7.4]	7.31 [7.12; 7.4]	**0.009**	0.089
HCO_3_^-^ (mmol/l); Med[IR]	19.7 [11.18; 24.0]	16.3 [7.6; 23.6]	**0.021**	0.082
pCO_2_ (mmHg); Med [IR]	32.0 [23.0; 38.0]	28.7 [18.05; 37.0]	**0.013**	0.087
BG (mmol/l); Med [IR]	24.35 [18.2; 30.8]	21.5 [17.0; 28.05]	**0.033**	0.071
HbA_1c_ (%); Med [IR]	11.6 [9.9; 13.7]	11.3, [9.8; 13.6]	0.957	0.002
C-peptide (ng/ml); Med [IR]	0.44 [0.25; 0.69]	0.57 [0.34; 1.15]	**<0.001**	0.149

Significant difference between groups in bold. Med, Median; IR, Interquartile range, 25. and 75. percentiles.

**Table 5:** Characteristics of the three subgroups regarding the parameters recorded at the presentation of T1DM.

Recorded parameter		Normal	Overweight	Obese	p	effect size (Ɛ^2^)	Post- hoc
**Age (years)**	Med [IR]	8.71 [4.83;11.98]	9.96 [6.27; 12.81]	9.04 [6.23; 11.62]	0.111	0.00	Ns
	n	776	92	54			
**pH**	Med [IR]	7.35 [7.23; 7.4]	7.29 [7.12; 7.39]	7.35 [7.2; 7.4]	**0.005**	0.01	Ow<Nw, Ob=Nw
	n	707	86	50			
**HCO_3_^-^ (mmol/l)**	Med [IR]	19.7 [11.18; 24.0]	15.3 [6.9; 22.7]	20.5 [10.85; 24.15]	**0.018**	0.01	Ow<Nw, Ob=Nw
	n	674	79	48			
**pCO_2_ (mmHg)**	Med [IR]	32.0 [23.0; 38.0]	27.5 [16.1; 36.83]	32.0 [21.05; 37.0]	**0.018**	0.01	Ow<Nw, Ob=Nw
	n	689	80	49			
**BG (mmol/l)**	Med [IR]	24.35 [18.2; 30.8]	21.45 [17.35; 28.18]	21.65 [14.63; 28.15]	0.090	0.00	Ns
	n	760	90	54			
**HbA_1c_ (%)**	Med [IR]	11.6 [9.9; 13.7]	11.7 [10.1; 13.6]	11.25 [9.35; 13.78]	0.567	0.00	Ns
	n	728	87	52			
**C-peptide (ng/ml)**	Med [IR]	0.44 [0.25; 0.69]	0.51 [0.3; 0.8]	0.71 [0.45; 1.7]	**< 0.001**	0.03	Ob>Nw, Ob>Ow, Nw=Ow
	n	679	79	50			

Significant difference between groups in bold. n= number of data. Med, Median; IR, Interquartile range, 25. and 75. percentiles. Nw, normal weight; Ow, overweight; Ob, obese, ns, nonsignificant.

### Subgroup analysis between two groups

Significant difference was found between ‘normal’ and ‘children with overweight’ regarding age at T1DM diagnosis (Z= -2.044, p=0.041, r=0.07), pH (Z= -2.597, p=0.009, r=0.09), HCO_3_^-^ (Z= -2.316, p=0.021, r=0.08), pCO_2_ (Z= -2.476, p=0.013, r=0.09), BG (Z= -2.132, p=0.033, r=0.07) and C-peptide levels (Z= -4.234, p<0.001, r=0.15). In normal-weight children, T1DM presented itself at a younger age than in children with overweight. Children with overweight found to have lower pH, HCO_3_^-^, pCO_2_ and BG as well as higher C-peptide levels than their normal-weight peers at the diagnosis of T1DM. No significant difference was found in HbA_1c_ levels (Z= -0.054, p=0.957, r=0.00) at presentation between the two subgroups. Detailed data are shown in **Table 4**.

C-peptide levels were independent of sex. The pH and HCO_3_^-^ levels were significantly different in females, but did not differ significantly in males. C-peptide differed significantly in two age groups (6-11.99; 12-17.99 yrs). In age group 0-5.99 years, no significant difference was found. Detailed data is shown in **Supplementary Material 1**.

### Subgroup analysis between the three groups

**Figure 1:**
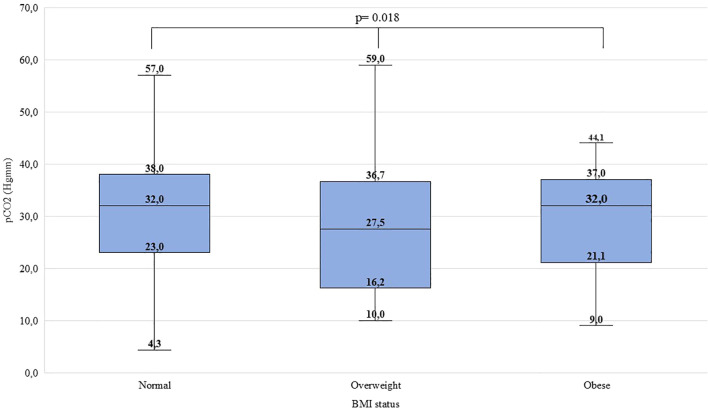
Distribution of pH at the manifestation of T1DM in the BMI groups. Medians, interquartile ranges, minimum and maximum values, and Kruskal-Wallis p-value are presented.

**Figure 2:**
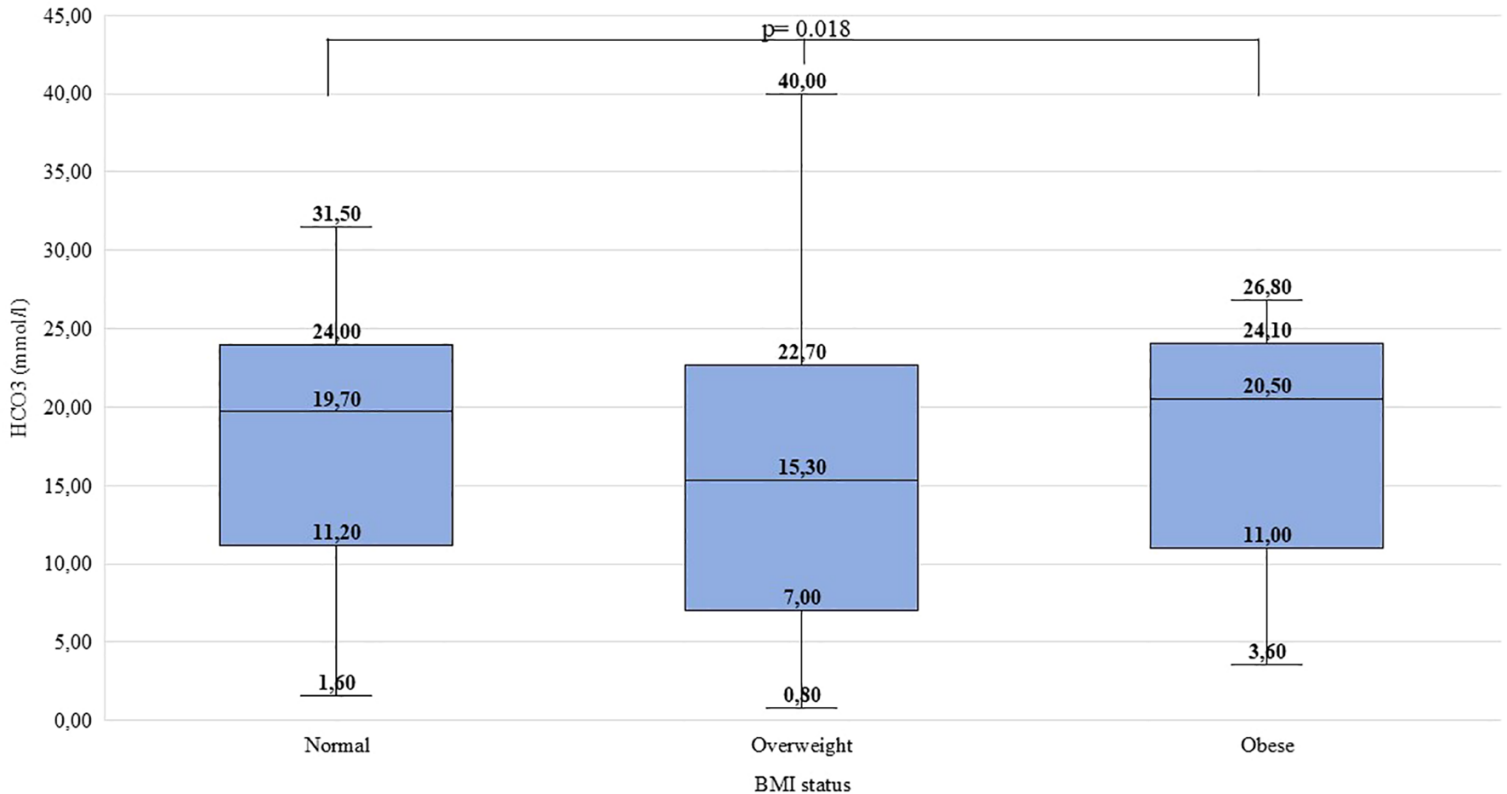
Distribution of HCO_3_^-^ levels (mmol/l) at the manifestation of T1DM in the BMI groups. Medians, interquartile ranges; minimum and maximum values, and Kruskal-Wallis p-value are presented.

**Figure 3:**
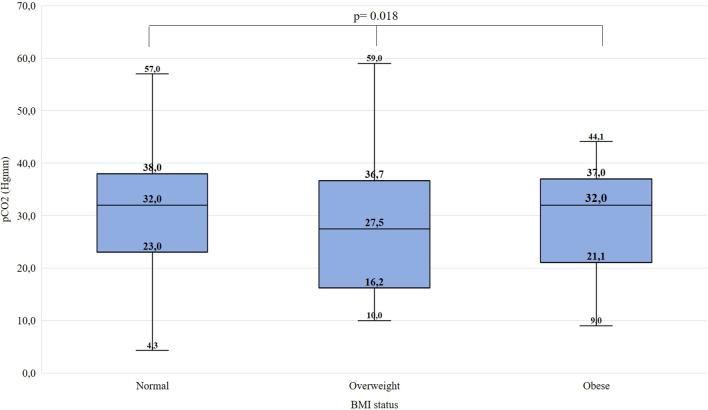
Distribution of partial pressure of CO_2_ at the manifestation of T1DM in the BMI groups. Medians, interquartile ranges; minimum and maximum values, and Kruskal-Wallis p-value are presented.

**Figure 4:**
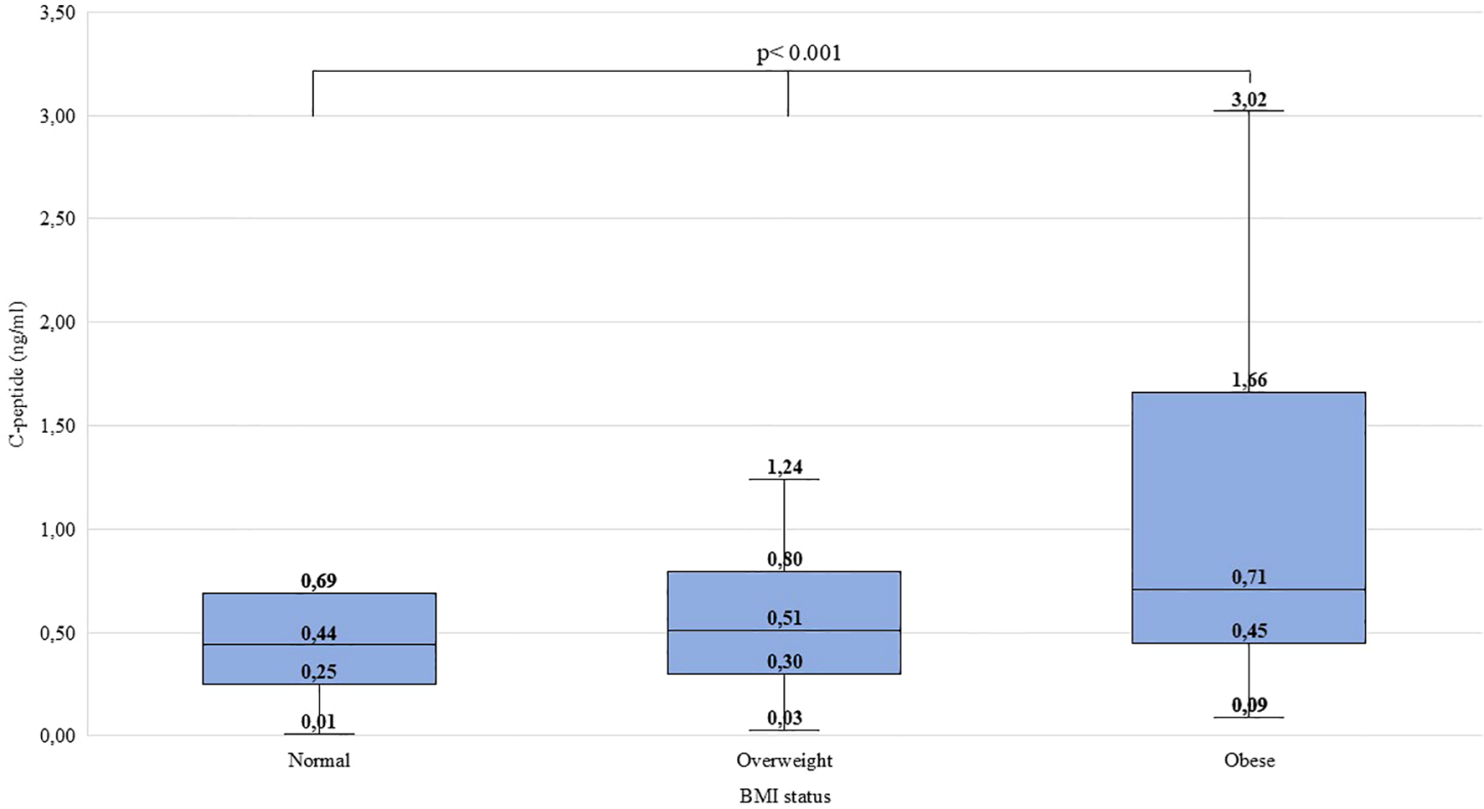
Distribution of C-peptide levels at the manifestation of T1DM in the BMI groups. Medians, interquartile ranges; minimum and maximum values, and Kruskal-Wallis p-value are presented.

Significant difference was found between ‘normal’, ‘overweight’, and ‘obese’ children regarding pH (χ^2^(2)=10.514, p=0.005, ϵ^2^ = 0.01), HCO_3_^-^ (χ^2^(2)=8.046, p=0.018, ϵ^2^ = 0.01), pCO_2_ (χ^2^(2)=7.988, p=0.018, ϵ^2^ = 0.01) and C-peptide levels (χ^2^(2)=25.684, p<0.001, ϵ^2^ = 0.03). Overweight children were found to have lower pH, lower HCO_3_^-^ and pCO_2_ than their normal-weight peers. Obese children had higher C-peptide levels than normal-weight and overweight children. Detailed data are shown in **Figures 1**–**4** and **Table 5**. No significant difference was found in age (χ^2^(2)=4.390, p=0.111, ϵ^2^ = 0.00), BG (χ^2^(2)=4.821, p=0.090, ϵ^2^ = 0.00), and HbA_1c_ levels (χ^2^(2)=1.136, p=0.567, ϵ^2^ = 0.00) at presentation of T1DM between the three subgroups.

C-peptide levels and pH differed significantly in both males and females. The HCO_3_^-^ levels differed significantly in females but did not differ significantly in males. The pH and C-peptide levels differed significantly in two age groups (6-11.99; 12-17.99 yrs). The HCO_3_^-^ and pCO2 levels differed significantly in age group 12-17.99 years. In the age group 0-5.99 years, no significant difference was found. Data is shown in **Supplementary Material 2**.

### DKA severity

We further analyzed the severity of DKA at presentation of T1DM in the subgroups; the data are shown in **Table 6**. The occurrence of DKA of any severity (no vs. mild, moderate, severe) was 41.8% in normal-weight, 59.3% among overweight, and 46.3% among obese children. Overweight and obese children had a significantly higher prevalence of DKA at presentation of T1DM compared to their normal-weight peers (χ^2^ (3,N = 903)=10.807, p=0.013). The occurrence of severe DKA (severe vs. no, mild, moderate) was 10.9% in normal-weight children, 27.5% in those who were overweight, and 16.7% in the obese group. Overweight and obese children had a significantly higher prevalence of severe DKA at presentation compared to their normal-weight peers (χ^2^ (3,N = 903)= 20.400, p < 0.001).

**Table 6:** Distribution of DKA in the subgroups.

DKA severity	Normal (n=758)	Overweight (n=91)	Obese (n=54)
No DKA, % (n)	58.2 (441_b_)	40.7 (37_a_)	53.7 (29_a,b_)
Mild DKA, % (n)	18.2 (138_a_)	17.6 (16_a_)	20.4 (11_a_)
Moderate DKA, % (n)	12.7 (96_a_)	14.3 (13_a_)	9.2 (5_a_)
Severe DKA, % (n)	10.9 (83_b_)	27.5 (25_a_)	16.7 (9_a,b_)
No and non-severe DKA, % (n)	89.1 (675)	72.6 (66)	83.3 (45)

Each subscript letter denotes a subset of BMI categories whose column proportions do not differ significantly from each other at the 0.05 level.

The presence and severity of DKA were further analyzed based on sex and age groups. Sex and age have no effect on the severity of DKA. In the 6-11.99-year age group, no association was found between BMI status and the severity of DKA. Data is shown in **Supplementary Material 3**.

## Discussion

To our knowledge, our study presents the first results about the differences in the presentation of T1DM in childhood depending on BMI status. In this retrospective cohort study involving 994 children with T1DM, we found lower pH, pCO_2_ and HCO_3_^-^ levels among those who were overweight, and higher C-peptide levels in children with obesity. Overall, children with overweight and obesity together had lower pH, pCO_2_ and HCO_3_^-^ parallel with higher BG and C-peptide levels than their normal-weight peers.

C-peptide levels were independent of sex. The pH and HCO_3_^-^ levels showed minimal differences regarding sex. Together with the median values in both groups (males and females) and with the effect sizes we can conclude that these slight differences between sexes have no clinical impact. Overall, sex has no significant effect on parameters at the onset of T1DM. Regarding age, grouping thresholds were based on the SWEET database; on the other hand, there are no physiological age thresholds, therefore, analyses based on age are of limited interpretability.

According to our results, children who are overweight are exposed to a more severe clinical presentation of T1DM. Weight loss, as a typical symptom of newly diagnosed T1DM, might mislead caregivers, considering it a positive factor. In addition, in the case of overweight and obesity, many associate it with type 2 diabetes mellitus (T2DM) and indicate lifestyle intervention. Therefore, the diagnosis of T1DM might be delayed. In our research, children with overweight were diagnosed in a worse condition (lower pH and HCO_3_^-^) than obese children.

Obese children exhibited higher C-peptide levels at the onset of T1DM, reflecting greater secretory capacity and a higher residual β-cell mass. Excess caloric intake promotes adipose tissue remodeling and macrophage infiltration, triggering inflammatory signaling that impairs insulin sensitivity. Concurrent lipid accumulation in skeletal muscle and hepatic insulin resistance further exacerbates metabolic dysregulation, resulting in compensatory hyperinsulinemia (17–20). In contrast to their higher insulin secretory capacity and higher residual β-cell function, children with overweight demonstrated a higher incidence of DKA at diagnosis of T1DM. Higher residual β-cell reserve indicates that the autoimmune process responsible for the development of T1DM destroyed fewer pancreatic β-cells. At the time of T1DM diagnosis, elevated insulin levels associated with a more severe metabolic condition indicate impaired insulin sensitivity, a process in which chronic obesity-related inflammation is thought to play a contributory role. Obesity, as mentioned above, means a chronic, low-grade inflammation. Chronic adipose tissue inflammation, infiltration, and activation of immune cells are key contributors to decreased insulin sensitivity. Insulin resistance is a metabolic condition in which the insulin-sensitive tissues (skeletal muscle, liver, adipose tissue) become less responsive to insulin action. As a response, β-cells increase their insulin secretion. Inflammatory mediators influence insulin signaling pathways, therefore affecting the secretion of insulin and modulating insulin resistance (21–23).

Obesity and weight management also play an essential role in the care of T1DM. Obesity alone increases the risk of cardiovascular morbidity and mortality. Patients with T1DM and subsequent overweight or obesity have an even higher risk for macro- and microvascular complications. Comprehensive care of children with T1DM should include effective weight management in addition to optimal glycemic targets (24). Key factors in weight loss are physical activity and diet. American Diabetes Association (ADA) recommends 60 minutes of moderate to vigorous intensity aerob activity daily for children with T1DM as well as for individuals with obesity. In terms of diet, there is no consistent guideline for children with T1DM and subsequent obesity. Following a Mediterranean diet (low glycemic index carbohydrates and low carbohydrate intake) might be helpful in optimal daily caloric intake according to ADA. On the other hand, children and adolescents with T1DM should not follow extreme carbohydrate-restricted diets (resulting in insufficient carbohydrate intake) due to the risk of DKA or hypoglycemia (25). Glucagon-like peptide-1 (GLP-1) receptor analogues are safe and effective in children with T2DM and obese children as well. To date, limited literature on pharmacological treatment with GLP-1 analogues in children with T1DM is available. Currently, no GLP-1 analogue is approved for children with T1DM and subsequent obesity (26, 27).

Our study has an impact on everyday clinical practice. In children with overweight, in case of significant or sudden weight loss can draw our attention or might be a red flag. In these cases, especially in a child with a previous unsuccessful weight loss history, detailed medical history and questioning are needed, as T1DM can be an underlying cause. Without a thorough medical history, overweight children may progress earlier into a more severe condition. With detailed anamnesis, severe DKA and a life-threatening condition might be avoided.

### Strengths

Our study represents data from a large cohort of T1DM children from the same center. Standardized clinical care and laboratory measurements were consistently provided to all children. To define overweight and obesity, we used the international WHO criteria. The latest ISPAD guideline (published in 2022) was used to categorize DKA in all cases.

### Limitations

Anthropometric data before the manifestation of T1DM in children are not known. As no recent data were available prior to the diagnosis of T1DM, we were unable to assess the degree of dehydration at the time of T1DM diagnosis. There is no measurement method to collect pre-diagnostic anthropometric data objectively. Although additional factors may influence BMI by the three-month follow-up, the literature supports that it correlates well with pre-diagnostic anthropometric parameters (7). As this measurement represented the first standardized assessment performed in all patients after T1DM diagnosis, it was selected for analysis. This supports our method using post-onset BMI Z-scores. As a retrospective study, missing values occurred in the recorded parameters, as shown in the related tables. Statistically significant differences associated with a lower effect size (except C-peptide comparisons). Age cannot be added properly to the statistical methods as a “confounder” because the age group thresholds are not sharply separated physiologically (like sex). Our study does not examine the additional factors that may have contributed to the development of DKA (28).

## Conclusion

In children with overweight and obesity, T1DM can develop as well. Children who were overweight or obese three months after T1DM diagnosis had lower pH, pCO_2_ and HCO_3_^-^ levels at the manifestation of T1DM. Children with overweight had higher C-peptide levels than normal-weight children. Higher C-peptide levels indicate higher insulin secretory capacity in children with overweight. This implies that T1DM manifests at an earlier phase, with a higher risk of DKA in children with overweight. This supports the hypothesis that obesity-related chronic inflammation contributes to decreased insulin sensitivity. Our findings support everyday clinical practice: when an overweight child presents with substantial weight loss, a comprehensive medical history is warranted, as T1DM may be an underlying cause. In case of weight loss in obese and overweight children, more attention should be paid to the classical symptoms of T1DM to diagnose it and prevent DKA. Severe DKA and a life-threatening condition might be avoided.

## Data Availability

The data analyzed in this study is subject to the following licenses/restrictions: The raw data supporting the conclusions of this article will be made available by the authors, without undue reservation. Requests to access these datasets should be directed to Eszter Muzslay, muzslay.eszter@semmelweis.hu.
