# Supplementary figures and images for: Overweight worsens the metabolic presentation of type 1 diabetes mellitus in children
Source: Front Endocrinol (Lausanne). 2026 Jan 9;16:1740046. doi: 10.3389/fendo.2025.1740046 (PMC12827166; doi:10.3389/fendo.2025.1740046)

## Slide 1
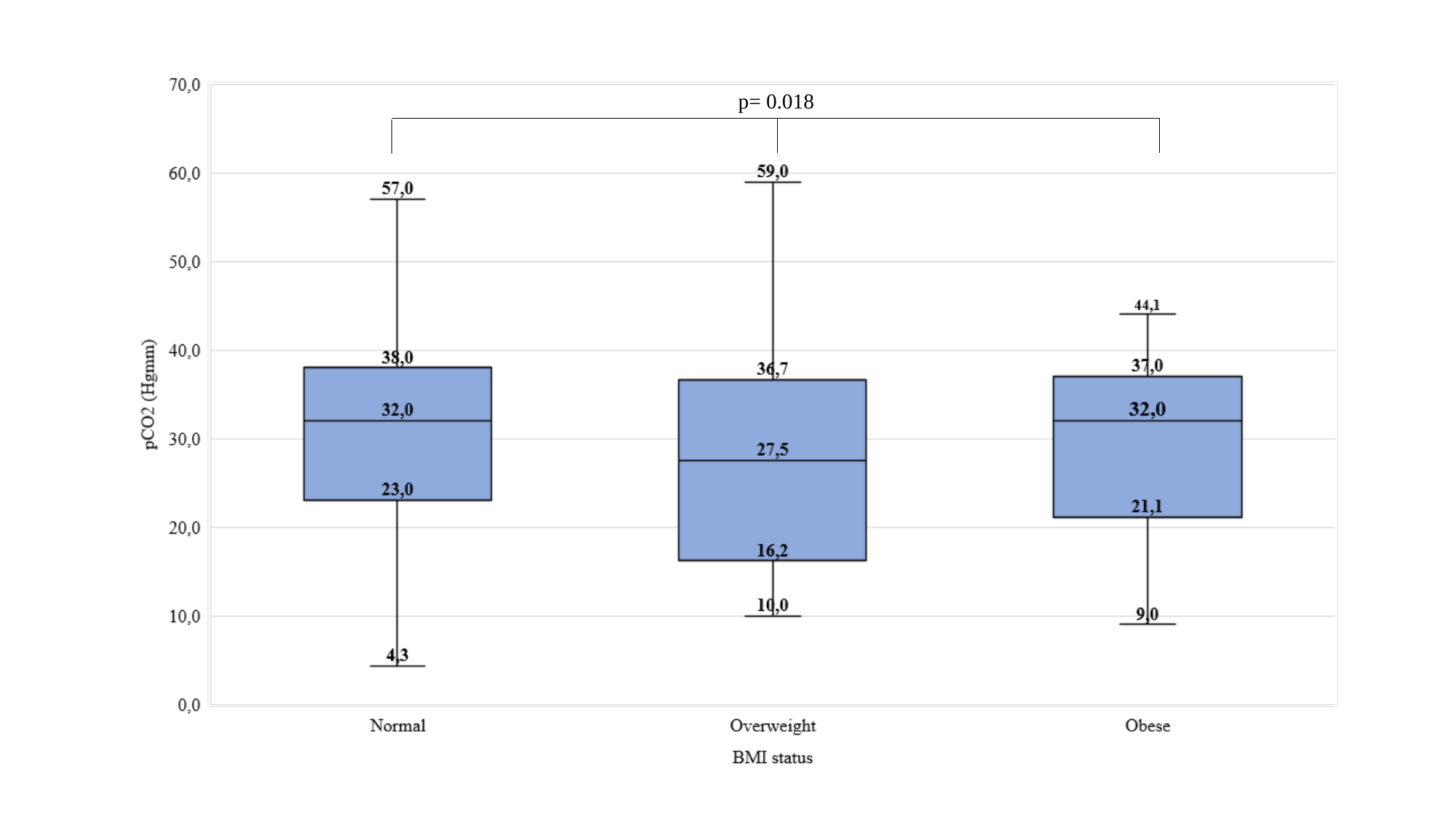

p= 0.018

## Slide 2
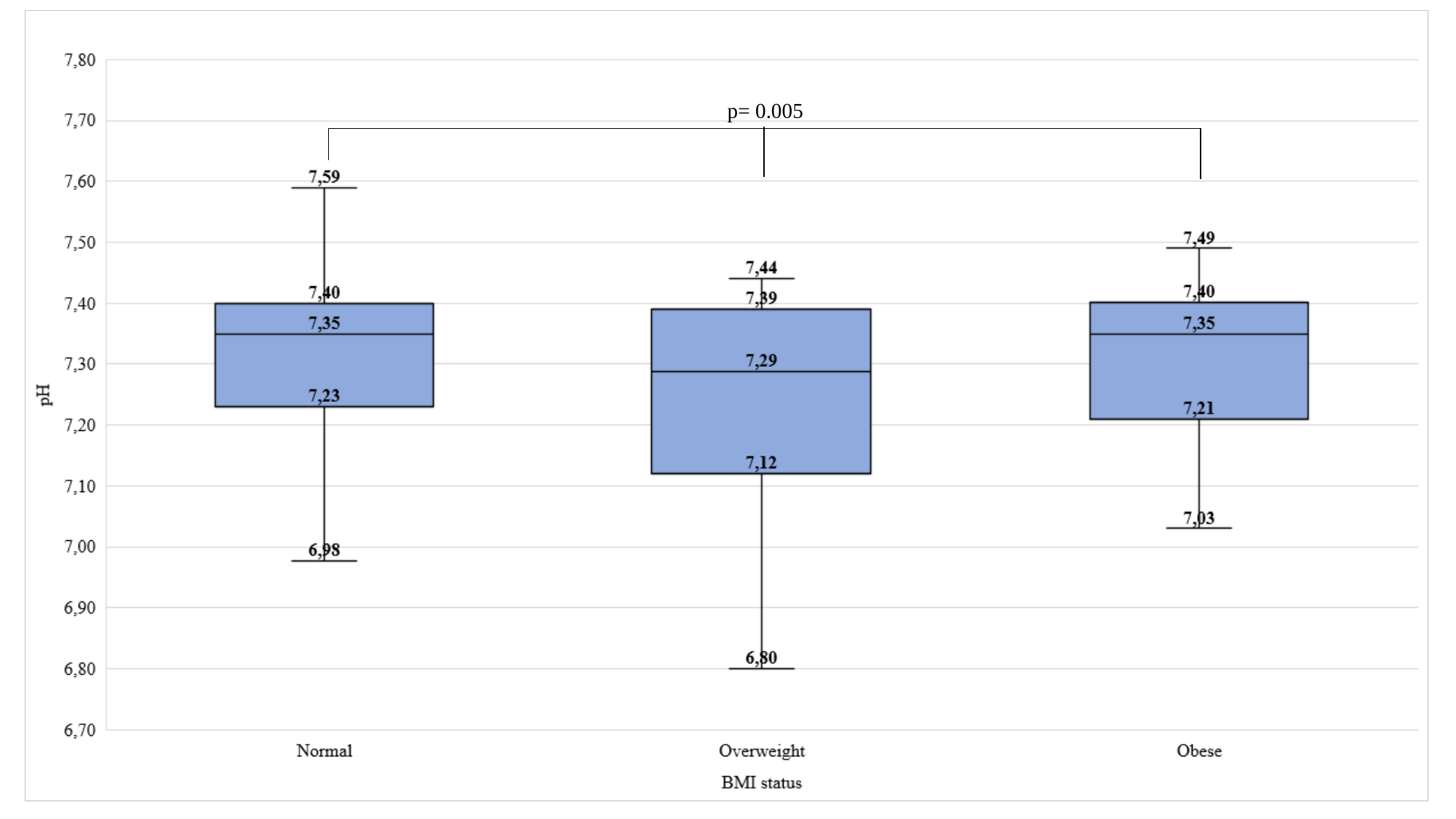

p= 0.005

## Slide 3
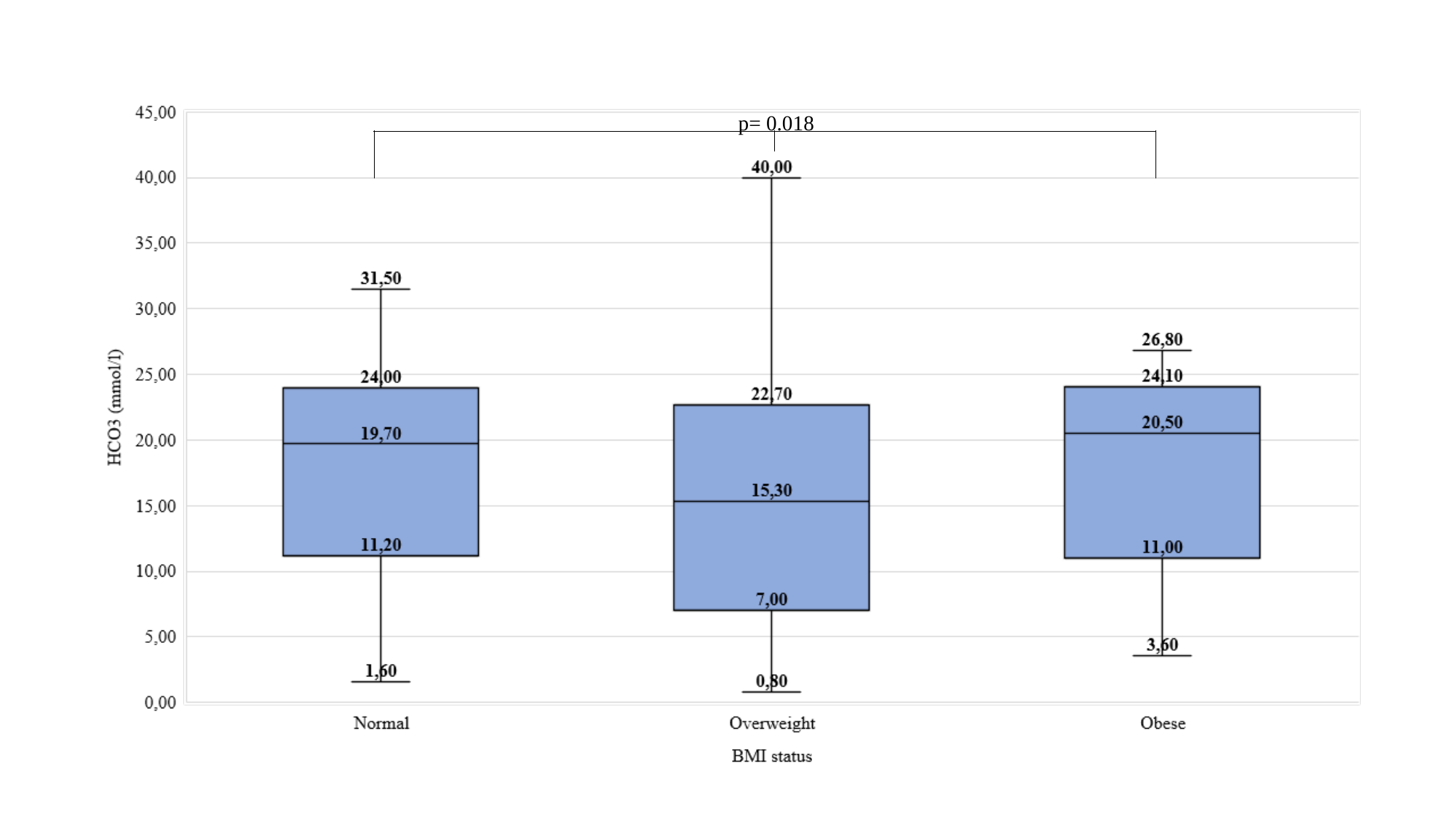

p= 0.018

## Slide 4
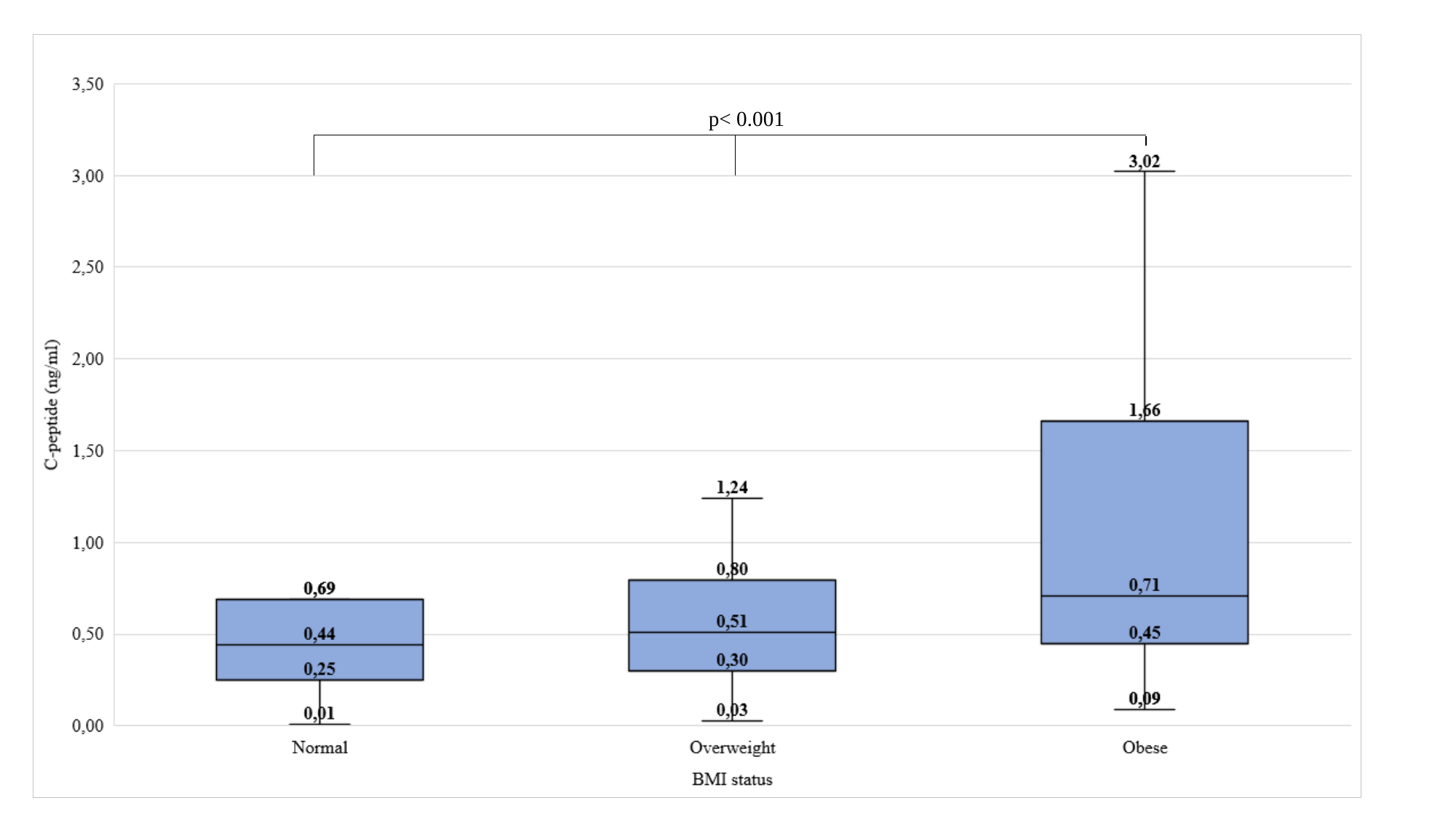

p< 0.001

Supplement: Supplementary file 1 [file Presentation1.pptx]
