# Supplementary material for: Overweight worsens the metabolic presentation of type 1 diabetes mellitus in children
Source: Front Endocrinol (Lausanne). 2026 Jan 9;16:1740046. doi: 10.3389/fendo.2025.1740046 (PMC12827166; doi:10.3389/fendo.2025.1740046)
Supplement: Supplementary file 2 [file Table1.docx]

**Supplementary Material 1. Comparisons of the BMI two subgroups by sex and age.**

**Comparisons of the BMI two subgroups by sex.**

| **Males** | **Normal** | **Children with overweight** | **p** | **r** |
| --- | --- | --- | --- | --- |
| Age (years); Med [IR], n=509 | 9.13 [4.83; 12.56] | 10.58 [7.13; 12.67] | 0.139 | 0.07 |
| pH; Med [IR], n=465 | 7.35 [7.23; 7.4] | 7.32 [7.15; 7.41] | 0.271 | 0.05 |
| HCO_3_^-^ (mmol/l); Med[IR], n=442 | 20.7 [11.7; 24.2] | 17.2 [9.0; 24.0] | 0.251 | 0.06 |
| pCO_2_ (mmHg); Med [IR], n=453 | 32.25 [24.0; 39.0] | 32.0 [19.0; 38.0] | 0.087 | 0.08 |
| BG (mmol/l); Med [IR], n=498 | 24.3 [17.4; 31.3] | 22.8 [16.2; 28.9] | 0.209 | 0.06 |
| HbA_1c_ (%); Med [IR], n=477 | 11.1 [9.8; 13.2] | 11.3 [9.45; 13.25] | 0.874 | 0.01 |
| C-peptide (ng/ml); Med [IR], n=446 | 0.44 [0.24; 0.69] | 0.60 [0.36; 1.09] | **< 0.001** | 0.17 |
| **Females** | **Normal** | **Children with overweight** | **p** | **r** |
| Age (years); Med [IR], n=413 | 8.04 [4.77; 11.33] | 9.0 [6.08; 11.17] | 0.2 | 0.06 |
| pH; Med [IR], n=378 | 7.36 [7.21; 7.4] | 7.31 [7.11; 7.39] | **0.008** | 0.14 |
| HCO_3_^-^ (mmol/l); Med [IR], n=359 | 19.1 [10.8; 23.4] | 14.7 [5.38; 22.63] | **0.026** | 0.12 |
| pCO_2_ (mmHg); Med [IR], n=365 | 31.0 [22.1; 36.6] | 27.55 [16.3; 36.93] | 0.055 | 0.10 |
| BG (mmol/l); Med [IR], n=406 | 24.5 [18.8; 30.55] | 20.7 [17.7; 27.85] | 0.061 | 0.09 |
| HbA_1c_ (%); Med [IR], n=390 | 12.0 [9.9; 14.0] | 11.7 [10.38; 14.0] | 1.000 | 0.00 |
| C-peptide (ng/ml); Med [IR], n=362 | 0.44 [0.27; 0.68] | 0.53 [0.31; 1.51] | **0.023** | 0.12 |

Med: Median, IR: Interquartile range, 25. and 75. percentiles, r: effect size measurement for Mann-Whitney U test.

**Comparisons of the BMI two subgroups by age.**

| **0-5.99 yrs** | **Normal** | **Children with overweight** | **p** | **r** |
| --- | --- | --- | --- | --- |
| Age (years); Med [IR], n=281 | 3.67 [2.33; 4.75] | 2.58 [1.71; 4.58] | 0.129 | 0.09 |
| pH; Med [IR], n=257 | 7.33 [7.2; 7.4] | 7.23 [7.06; 7.39] | 0.064 | 0.12 |
| HCO_3_^-^ (mmol/l); Med[IR], n=244 | 17.0 [9.08; 22.83] | 14.85 [6.05; 21.7] | 0.201 | 0.08 |
| pCO_2_ (mmHg); Med [IR], n=246 | 29.0 [19.7; 36.0] | 24.8 [14.18; 34.15] | 0.125 | 0.10 |
| BG (mmol/l); Med [IR], n=276 | 25.45 [19.6; 32.45] | 23.1 [17.2; 29.45] | 0.353 | 0.06 |
| HbA_1c_ (%); Med [IR], n=258 | 10.7 [9.3; 12.2] | 10.6 [9.6; 11.8] | 0.82 | 0.01 |
| C-peptide (ng/ml); Med [IR], n=229 | 0.29 [0.17; 0.48] | 0.3 [0.19; 0.44] | 0.946 | 0.00 |
| **6-11.99 yrs** | **Normal** | **Children with overweight** | **p** | **r** |
| Age (years); Med [IR], n=404 | 9.08 [7.56; 10.5] | 9.13 [7.54; 10.5] | 0.655 | 0.02 |
| pH; Med [IR], n=370 | 7.36 [7.25; 7.41] | 7.32 [7.15; 7.41] | 0.119 | 0.08 |
| HCO_3_^-^ (mmol/l); Med[IR], n=352 | 19.7 [13.33; 23.9] | 15.4 [6.35; 23.98] | **0.039** | 0.11 |
| pCO_2_ (mmHg); Med [IR], n=364 | 31.0 [24.0; 37.0] | 28.35 [19.93; 36.0] | 0.051 | 0.10 |
| BG (mmol/l); Med [IR], n=393 | 23.9 [17.0; 30.0] | 20.95 [17.0; 27.88] | 0.217 | 0.06 |
| HbA_1c_ (%); Med [IR] n=378 | 11.85 [10.3; 14.0] | 11.6 [9.78; 13.33] | 0.28 | 0.06 |
| C-peptide (ng/ml); Med [IR], n=359 | 0.43 [0.28; 0.66] | 0.56 [0.4; 0.94] | **< 0.001** | 0.175 |
| **12-17.99 yrs** | **Normal** | **Children with overweight** | **p** | **r** |
| Age (years); Med [IR], n=237 | 13.75 [12.75; 15.17] | 13.75 [12.83; 15.0] | 0.989 | 0.014 |
| pH; Med [IR], n=216 | 7.36 [7.24; 7.4] | 7.31 [7.16; 7.39] | 0.097 | 0.113 |
| HCO_3_^-^ (mmol/l); Med[IR], n=205 | 21.75 [12.8; 24.8] | 17.5 [9.1; 24.15] | 0.192 | 0.091 |
| pCO_2_ (mmHg); Med [IR], n= 208 | 36.0 [25.0; 41.35] | 33.6 [19.0; 38.0] | **0.042** | 0.141 |
| BG (mmol/l); Med [IR], n=235 | 23.8 [17.95; 30.1] | 21.05 [16.85; 27.65] | 0.296 | 0.068 |
| HbA_1c_ (%); Med [IR], n=231 | 12.5 [10.4; 14.0] | 12.65 [10.18; 14.03] | 0.961 | 0.003 |
| C-peptide (ng/ml); Med [IR], n=220 | 0.69 [0.48; 1.01] | 0.86 [0.53; 1.74] | **0.033** | 0.144 |

Med: Median, IR: Interquartile range, 25. and 75. percentiles, r: effect size measurement for Mann-Whitney U test.

**Supplementary material 2. Comparisons of the BMI three subgroups by sex and age.**

**Comparisons of the BMI three subgroups by sex.**

| **Males** | **Normal (n=428)** | **Overweight (n=53)** | **Obese (n=28)** | **p** | **ε^2^** |
| --- | --- | --- | --- | --- | --- |
| Age (years); Med [IR] | 9.13 [4.83; 12.56] | 11.17 [6.55; 13.34] | 9.75 [7.33; 11.39] | 0.195 | 0.00 |
| pH; Med [IR] | 7.35 [7.23; 7.4] | 7.29 [7.13; 7.4] | 7.37 [7.26; 7.42] | **0.045** | 0.01 |
| HCO_3_^-^ (mmol/l); Med [IR] | 20.7 [11.7; 24.2] | 16.1 [8.0; 23.7] | 21.7 [12.68; 24.43] | 0.322 | 0.00 |
| pCO_2_ (mmHg); Med [IR] | 32.25 [24.0; 39.0] | 29.6 [16.45; 38.0] | 33.0 [26.35; 36.2] | 0.148 | 0.00 |
| BG (mmol/l); Med [IR] | 24.3 [17.4; 31.3] | 23.5 [17.0; 30.2] | 19.8 [13.73; 26.35] | 0.096 | 0.01 |
| HbA_1c_ (%); Med [IR] | 11.1 [9.8; 13.2] | 11.25 [9.4; 13.23] | 11.3 [9.7; 13.4] | 0.975 | 0.00 |
| C-peptide (ng/ml); Med [IR] | 0.44 [0.24; 0.69] | 0.54 [0.30; 0.95] | 0.73 [0.58; 1.37] | **< 0.001** | 0.04 |
| **Females** | **Normal (n=348)** | **Overweight (n=39)** | **Obese (n=26)** | **p** | **ε^2^** |
| Age (years); Med [IR] | 8.04 [4.77; 11.33] | 9.08 [6.17; 10.83] | 8.96 [6.06; 12.89] | 0.399 | 0.00 |
| pH; Med [IR] | 7.36 [7.21; 7.4] | 7.24 [7.11; 7.39] | 7.33 [7.12; 7.4] | **0.025** | 0.01 |
| HCO_3_^-^ (mmol/l); Med [IR] | 19.1 [10.8; 23.4] | 11.8 [4.85; 21.6] | 19.0 [6.4; 24.05] | **0.029** | 0.01 |
| pCO_2_ (mmHg); Med [IR] | 31.0 [22.0; 36.6] | 26.8 [14.38; 33.25] | 28.8 [16.65; 30.4] | 0.079 | 0.01 |
| BG (mmol/l); Med [IR] | 24.5 [18.8; 30.55] | 20.3 [18.0; 26.3] | 24.35 [16.65; 30.4] | 0.078 | 0.01 |
| HbA_1c_ (%); Med [IR] | 12.0 [9.9; 14.0] | 12.4 [10.85; 14.0] | 10.9 [9.0; 14.0] | 0.19 | 0.00 |
| C-peptide (ng/ml); Med [IR] | 0.44 [0.27; 0.68] | 0.45 [0.3; 0.74] | 0.66 [0.38; 2.27] | **0.012** | 0.02 |

Med: Median, IR: Interquartile range, 25. and 75. percentiles, ε^2^: epsilon-squared effect size measurement for Kruskal-Wallis H test.

**Comparisons of the BMI three subgroups by age.**

| **0-5.99 years** | **Normal** | **Overweight** | | **Obese** | | **p** | **ε^2^** |
| --- | --- | --- | --- | --- | --- | --- | --- |
| Age (years); Med [IR] | 3.67 [2.33; 4.75] | 2.5 [1.77; 3.94] | | 4.17 [1.5; 5.25] | | 0.089 | 0.01 |
| pH; Med [IR] | 7.33 [7.2; 7.4] | 7.17 [7.05; 7.39] | | 7.33 [7.12; 7.4] | | 0.141 | 0.01 |
| HCO_3_^-^ (mmol/l); Med [IR] | 17.0 [9.08; 22.83] | 13.4 [5.3; 21.4] | | 16.6 [6.3; 22.6] | | 0.409 | 0.00 |
| pCO_2_ (mmHg); Med [IR] | 29.0 [19.7; 36.0] | 23.0 [14.35; 35.15] | | 27.0 [13.0; 34.2] | | 0.298 | 0.00 |
| BG (mmol/l); Med [IR] | 25.45 [19.6; 32.45] | 23.1 [18.4; 31.7] | | 22.8 [12.55; 28.75] | | 0.533 | 0.00 |
| HbA_1c_ (%); Med [IR] | 10.7 [9.3; 12.2] | 10.85 [10.1; 12.18] | | 10.5 [8.55; 11.55] | | 0.539 | 0.00 |
| C-peptide (ng/ml); Med [IR] | 0.29 [0.17; 0.48] | 0.28 [0.17; 0.37] | | 0.41 [0.19; 0.77] | | 0.349 | 0.00 |
| **6-11.99 years** | **Normal** | **Overweight** | | **Obese** | | **p** | **ε^2^** |
| Age (years); Med [IR] | 9.08 [7.56; 10.5] | 9.33 [7.94; 10.38] | | 8.96 [7.38; 10.6] | | 0.787 | 0.00 |
| pH; Med [IR] | 7.36 [7.25; 7.41] | 7.32 [7.15; 7.4] | | 7.32 [7.14; 7.41] | | **0.263** | 0.00 |
| HCO_3_^-^ (mmol/l); Med [IR] | 19.7 [13.33; 23.9] | 15.5 [5.95; 22.7] | | 14.6 [6.5; 24.2] | | 0.102 | 0.01 |
| pCO_2_ (mmHg); Med [IR] | 31.0 [24.0; 37.0] | 28.7 [16.4; 35.0] | | 28.0 [21.0; 36.0] | | 0.145 | 0.01 |
| BG (mmol/l); Med [IR] | 23.9 [17.0; 30.0] | 20.8 [17.1; 27.65] | | 21.35 [15.6; 29.73] | | 0.463 | 0.00 |
| HbA_1c_ (%); Med [IR] | 11.85 [10.3; 14.0] | 12.1 [9.88; 13.3] | | 11.05 [9.7; 13.4] | | 0.334 | 0.00 |
| C-peptide (ng/ml); Med [IR] | 0.43 [0.28; 0.66] | 0.50 [0.34; 0.61] | | 0.64 [0.45; 1.53] | | **< 0.001** | 0.04 |
| **12-17.99 years** | **Normal** | | **Overweight** | | **Obese** | **p** | **ε^2^** |
| Age (years); Med [IR] | 13.75 [12.75; 15.17] | | 13.54 [12.56; 15.0] | | 13.75 [13.08; 15.42] | 0.585 | 0.00 |
| pH; Med [IR] | 7.36 [7.24; 7.4] | | 7.24 [7.14; 7.38] | | 7.38 [7.33; 7.4] | **0.02** | 0.03 |
| HCO_3_^-^ (mmol/l); Med [IR] | 21.75 [12.8; 24.8] | | 13.5 [7.6; 23.5] | | 23.2 [20.58; 25.28] | **0.035** | 0.02 |
| pCO_2_ (mmHg); Med [IR] | 36.0 [25.0; 41.35] | | 28.1 [17.13; 37.75] | | 37.0 [35.3; 40.7] | **0.022** | 0.03 |
| BG (mmol/l); Med [IR] | 23.8 [17.95; 30.1] | | 21.1 [17.2; 27.8] | | 19.8 [13.9; 25.0] | 0.391 | 0.00 |
| HbA_1c_ (%); Med [IR] | 12.5 [10.4; 14.0] | | 12.1 [10.3; 14.0] | | 12.9 [9.3; 14.7] | 0.905 | 0.00 |
| C-peptide (ng/ml); Med [IR] | 0.69 [0.48; 1.01] | | 0.77 [0.44; 1.34] | | 1.49 [0.73; 2.31] | **0.013** | 0.03 |

Med: Median, IR: Interquartile range, 25. and 75. percentiles, ε^2^: epsilon-squared effect size measurement for Kruskal-Wallis H test.
